# Magnesium Silicate Honeycomb Structure on Attapulgite Clay Composite by Self-Template Method for Adsorption of Methylene Blue

**DOI:** 10.3390/ma18040792

**Published:** 2025-02-11

**Authors:** Tin Kyawoo, Tiefeng Wei, Naveed Karim, Chao Jiang, Saeed Ahmed, Huiyu Li, Yongjun Feng

**Affiliations:** 1 State Key Laboratory of Chemical Resource Engineering, College of Chemistry, Beijing University of Chemical Technology, No. 15 Beisanhuan East Road, Beijing 100029, China; tinkyawoo.mu@gmail.com (T.K.); karim@buct.edu.cn (N.K.); 15222443969@163.com (C.J.); huiyuli@buct.edu.cn (H.L.); 2 Guangxi Petrochemical Company, PetroChina, Qinzhou 535008, China; weitf@petrochina.com.cn; 3 Department of Chemistry, University of Chakwal, Chakwal 48800, Pakistan

**Keywords:** adsorption, hydrothermal method, magnesium silicate, methylene blue, surface modification

## Abstract

A series of porous silica materials coated with honeycomb-like magnesium silicate were prepared under hydrothermal conditions, using natural one-dimensional porous attapulgite as the template and a silicon source with different Mg/Si ratios, by adjusting the amount of MgCl_2_⋅6H_2_O and the attapulgite precursor and regulating pH. The influence of the Mg/Si ratios was carefully investigated on morphology, pore structure, and related adsorption actions toward methylene blue. The Langmuir isotherm and pseudo-second-order kinetic models were used to explain the adsorption behavior of methylene blue. The synthesized composite with the lowest magnesium content displayed the highest removal capability of 166.67 mg/g for methylene blue, with a zeta potential of −18.18 mV, a specific surface area of 310.4 m^2^/g, and an average pore size of 3.7 nm. The removal result was the synergetic adsorption between porous magnesium silicate grown on the surface and the rest of the silica, further indicating that the attapulgite is available as a silicon source and a rod-shaped template for magnesium silicate.

## 1. Introduction

The harmful effects of high dye concentrations in water on aquatic and human life make them a major concern [1]. There are several different technologies available to eliminate fabric dyes from water. Among them, adsorption is one of the most versatile and promising techniques because it is inexpensive, easy to operate, simple and adaptable in design, and does not produce secondary pollutants [2]. Recently, natural clays, including magnesium silicates, have become attractive as high-performance adsorbents because of their affordability, environmental friendliness, high porosity, and large surface area [3,4]. A metal hydroxide octahedral sheet with a tetrahedral-to-octahedral ratio of 2:1 (T-O-T) or 1:1 (T-O) holds one or two silica tetrahedral sheets, which together form the layered structure of silicate minerals. An octahedral sheet is made up of Al_2_(OH)_6_ units, in which Al can be substituted with other metal ions, like Mg, Fe, and so forth, whereas a tetrahedral sheet is typically composed of Si_2_O_6_(OH)_4_ units [5]. Some structures of a few magnesium silicates are sepiolite, 2:1 phyllosilicates/talc, and 1:1 phyllosilicate/serpentine group [6,7]. Silicate clays are used in a wide range of fields, including water treatment, due to their layer structure, silanol and metal hydroxyl groups, and ion exchangeability [8].

Furthermore, magnesium silicate’s adsorption performance mainly depends on the pore structure and surface properties, which are related to the corresponding synthesis methods [9]. For example, Huang et al. prepared a group of silicate hydrates with an improved adsorption performance toward methylene blue following negative surface charge density. Attapulgite is one type of natural clay made of nanoscale building blocks of hydrated magnesium silicate [10], with the chemical formula of Mg_5_Si_8_O_20_(OH)_2_(OH_2_)_4_⋅4H_2_O, which is a kind of one-dimensional mineral ranging in diameter from 20 to 70 nm and length from 1 to 5 µm [11]. Generally, attapulgite has been used as an adsorbent toward heavy metals and organic dyes due to its low cost and porous structure [12,13]. Yet, the removal capacity is limited. It is necessary to modify the pore structure and surface properties, which may enhance the removal performance and then accelerate the removal rate. Natural clay with high adsorption performance can be achieved through organic modification, pillaring, and acid treatment [14,15,16,17]. Anirudhan and Ramachandran [18] modified bentonite clay with hexadecyl trimethyl ammonium chloride to enhance the adsorption of some basic dyes from aqueous solutions, such as crystal violet and methylene blue. Furthermore, it is possible to create porous silicate composite materials and porous silica nanorods from naturally occurring palygorskite [19,20]. The formation, pore structure, and associated removal performance toward contaminants can be optimized by appropriately adjusting the feeding ratios of Mg/Si precursors (later, abbreviated as Mg/Si ratio) in the reaction system [9,21].

Until now, various hard or soft templates have been primarily used to develop layered magnesium silicates with improved pore structures [19,22]. However, removing templates is tricky, energy-intensive, and not discerning [23]. The simple synthesis design and self-templating methods are gaining attention as a simple way to overcome the difficulty to remove the used templates [24]. The distinct characteristics of natural clays, including easy availability, low cost, high specific surface area, and wide-ranging structure, make them a desirable class of template materials [25,26]. Among the available templates, attapulgite is regarded as a competitive template material used to create various nanomaterials [20].

**Figure 1:**
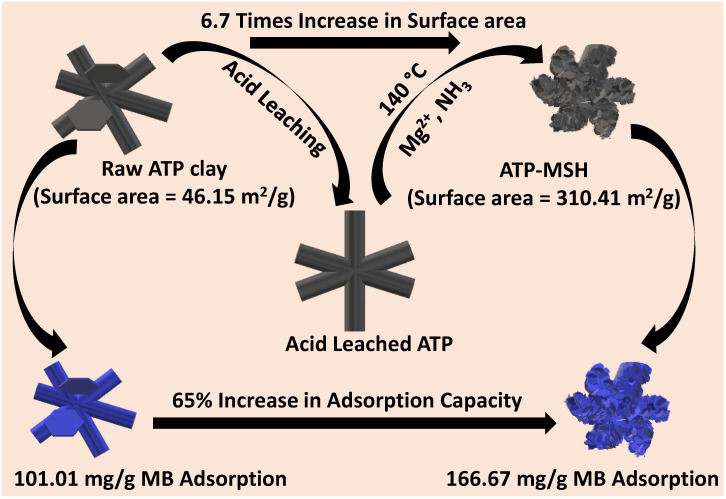
Fabrication and adsorption behavior of synthesized honeycomb ATP-MSH composites.

In this work, we develop a series of silica coated with magnesium silicate with different Mg/Si ratios using the silica precursor as a Si-bearing self-templating agent, derived from raw attapulgite minerals under hydrothermal conditions. The primary objective is to alter the natural clay to improve its adsorption capacity for cationic dye polluters. The adsorption behavior of the composites toward methylene blue dye is carefully investigated. Figure 1 demonstrates the formation of the honeycomb structure of the ATP-MSH composite with the influence of Mg/Si ratios on the surface area and corresponding adsorption capacity toward methylene blue compared with raw clay.

## 2. Experimental Section

### 2.1. Materials and Method

All the chemicals, including ammonia solution (65% vol), ammonium chloride, hydrochloric acid solution (36% *w*/*w*), magnesium chloride hexahydrate, and methylene blue (MB), were acquired from Sigma Aldrich(Shanghai) Trading Co., Ltd. (Shanghai, China) and were of analytical quality. Attapulgite (ATP) was obtained from Gansu Western Attapulgite Research and Application Institute (Baiyin, China).

A series of silica porous materials coated with magnesium silicate were produced through the process of acid leaching of raw attapulgite minerals (R-ATP). Then, a hydrothermal reaction was performed [20]. When treating acid leaching, 3.0 g of R-ATP was dispersed into 60 mL of HCl solution with a 4.0 mol/L concentration to ensure a unified suspension. The suspension was put into a 100 mL stainless-steel reaction vessel and placed in an oven at 140 °C for 6 h. After allowing the vessel to naturally cool to room temperature, the precipitate was separated by centrifugation, washed with deionized water, dried for 12 h at 80 °C in an oven, and labeled as H-ATP. Four magnesium silicate hydrate (MSH) composites with different Mg/Si mole ratios (0.67–1.5) were produced by the hydrothermal method. For MSH with Mg/Si = 0.67, 20 mmol (1.21 g) of H-ATP was mixed with 60 mL of water containing 13.34 mmol (2.7120 g) of MgCl_2_⋅6H_2_O, 1.605 g (30 mmol) of NH_4_Cl, and 3 mL of 65% ammonia solution. After 20 min of sonication, it was removed into a 100 mL stainless-steel autoclave reactor and heated to 140 °C for 12 h. The white product was centrifuged, washed with deionized water, and allowed to cool to room temperature before being dried for 12 h at 80 °C. For other MSH samples, the same process was carried out, and the resulting products were labeled as ATP-MSH-0.67 to ATP-MSH-1.50 according to Mg/Si ratios, respectively.

### 2.2. Instrumentation

An X-ray diffractometer (SHIMADZU XRD-6000, Tokyo, Japan) was used to analyze the crystal structure at 40 kV using Cu Kα radiation (λ = 1.5406 Å). Using an FT-IR Spectrophotometer (SHIMADZU IRspirit-A224160, Tokyo Japan), the functional groups were examined in the range of 4000–400cm^−1^. The surface morphologies were examined using SEM (Zeiss Supra 55, Oberkochen, Germany). Low-temperature nitrogen adsorption–desorption tests were conducted using a BET analyzer (BEISHIDE BSD-PM2, Beijing, China) after a three-hour degassed treatment at 120 °C. A TGA-DTG analyzer (SDT Q-600, TA, Beijing, China) with a sensitivity of 0.1 μg and 0.001 °C was used to perform a thermal decomposition test. A Malvern Zeta sizer Pro (Malvern Panalytical, Malvern, UK) was used to measure the zeta potential.

### 2.3. Adsorption Experiment

For adsorption kinetics, 50.0 mg of each composite was dispersed into 100 mL (100.0 mg/L) of methylene blue at 25 °C and set in a thermostat shaker with a shaking speed of 150 rpm. A wavelength of 664 nm was used to perform spectrophotometric analysis on the dye concentrations after separating the solid adsorbent by centrifugation. Equation (1) was used to determine the adsorption capacity with time (*q_t_*):

(1) $$q_{t}=\frac{\left(C_{0}-C_{t}\right)\;mg/L\times volume\;of\;solution\;(L)}{mass\;of\;adsorbent\;(g)}$$

where *q_t_* is the adsorption capacity at time *t* (mg/g), *C*_0_ is the initial concentration of dye (mg/L), and *C_t_* is the concentration of dye at time *t* (mg/L). Furthermore, the removal rate of the adsorption reaction was calculated using the pseudo-first- and pseudo-second-order kinetic models in Equations (2) and (3):

(2) $$\ln\left(q_{e}-q_{t}\right)=lnq_{e}-k_{1}t$$

(3) $$\frac{t}{q_{t}}=\frac{1}{k_{2}q_{e}^{2}}+\frac{t}{q_{e}}$$

where *q_e_* (mg/g) and *q_t_* (mg/g) are the respective amounts of dye adsorbed at equilibrium and at time *t* (h), and *k*_1_ (h^−1^) and *k*_2_ (mgg^−1^ h^−1^) are the respective rate constants for the pseudo-first- and pseudo-second-order models.

The Elovich kinetic model is described in Equation (4):

(4) $$q_{t}=\frac{1}{\alpha }\;Ln\left(\alpha \beta \right)+\frac{1}{\beta }\;Ln(t)$$

where the constant *α* is the initial adsorption rate (mg g^−1^ min^−1^) and *β* (g mg^−1^) is related to the activation energy and the adsorption heat.

Then, 50 mg of adsorbent was dispersed into individual 100.0 mL MB solutions with different concentrations ranging from 80.0 mg/L to 300.0 mg/L for the isotherm investigation. After the equilibrium time, the remaining dye concentrations were examined, and the equilibrium adsorption amount was calculated using Equation (5). The Langmuir model in Equation (6) and the Freundlich model in Equation (7) were plotted using the obtained data:

(5) $$q_{e}=\frac{\left(C_{0}-C_{e}\right)\;mg/L\times volume\;of\;solution\;(L)}{mass\;of\;adsorbent\;(g)}$$

(6) $$\frac{C_{e}}{q_{e}}=\frac{C_{e}}{q_{m}}+\frac{1}{K_{L}q_{m}}$$

(7) $$logq_{e}=logK_{F}+\frac{1}{n}logC_{e}$$

where *C_e_* (mg/L) is the equilibrium dye concentration, *q_e_* (mg/g) is the amount of dye adsorbed at the equilibrium state, *K_L_*, *K_F_*, and *n* are the empirical constants, and *q_m_* (mg/g) is the maximum adsorption capacity toward dye.

## 3. Results and Discussion

### 3.1. Structure and Chemical Composition

**Figure 2:**
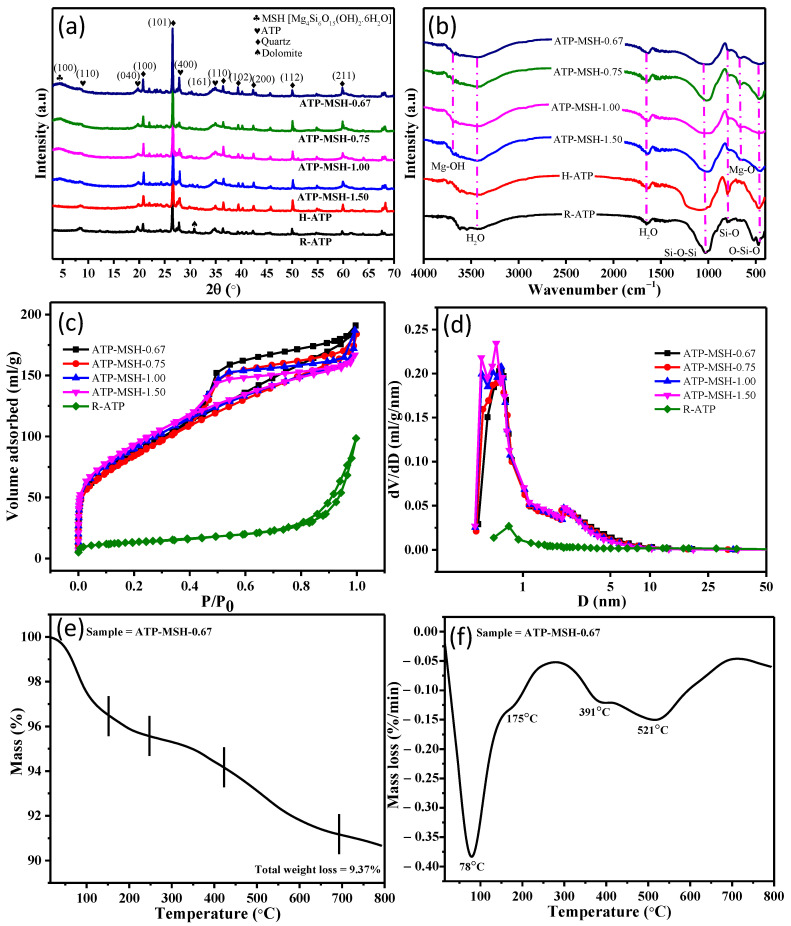
(**a**) XRD patterns, (**b**) FT-IR spectra, (**c**) nitrogen adsorption–desorption isotherm, (**d**) pore size distributions, (**e**) TGA curves, and (**f**) DTG curves for thermal degradation of composites with different Mg/Si ratios.

Figure 2a displays the crystal nature of raw ATP (R-ATP), acid-leached ATP (H-ATP), and a series of composites with different Mg/Si ratios. The XRD spectrum of R-ATP exhibits the characteristic diffractions of attapulgite (ATP) clay at 2θ = 8.44°, 19.68°, 27.8°, and 34.9° for (110), (040), (400), and (161) crystal planes, respectively [27,28,29]. Moreover, R-ATP also displays the diffraction pattern of quartz at 2θ = 20.76°, 26.52°, 36.44°, 39.34, 42.3°, 49.98°, and 59.86° for (100), (101), (110), (102), (200), (112), and (211) crystal planes (JCPDS Card No. 46-1045) and the diffraction pattern of dolomite at 2θ = 30.78° (JCPDS Card No. 36-0426), indicating raw clay is composed of ATP, quartz, and dolomite. After hydrochloric acid treatment, dolomite was completely removed from the raw material, and weak diffraction peaks of ATP were still observed, indicating that ATP has a high resistance to hydrochloric acid. Although ATP was not entirely removed from the raw material, the emergence of a strong silica diffraction peak between 2θ = 20 and 30° revealed the successful extraction of silica from raw ATP [30]. The silica diffraction peak’s intensity gradually dropped with an increase in Mg loading. The resulting magnesium silicate hydrates (MSH) showed similar peaks of sepiolite type MSH, Mg_4_Si_6_O_15_(OH)_2_.6H_2_O (JCPDS Card No. 29-1492) at 2θ = 4.5°, 20.12°, 34.62°, and 60.89°, which corresponded to the (100), (310), (102), and (4 15 1) crystal planes, excluding a slight shift of the (100) diffraction plane.

Figure 2b shows FT-IR spectra of raw ATP (R-ATP), acid-leached ATP (H-ATP), and a series of the prepared composites. The adsorbed and structural water in silica and magnesium silicate exhibited broad absorption bands around 3433 cm^−1^ and 1640 cm^−1^. H-ATP displayed a broad absorption band at 1094 cm^−1^ associated with the stretching vibration of Si-O-Si, an absorption band at 463 cm^−1^ related to the bending vibration of O-Si-O, and an additional absorption band centered at 798 cm^−1^ for Si-O vibration, which had more significant intensity compared to other MSH composites [31,32,33]. In comparison, the FT-IR spectra of composites were similar to H-ATP, except for a few typical absorption bands shifting to a lower wavenumber. For instance, the absorption band centered at 798 cm^−1^ for the Si-O vibration and the broad absorption band at roughly 1094 cm^−1^ associated with the stretching vibration of Si-O-Si were shifted to 778 cm^−1^ and 1008 cm^−1^, respectively. Additionally, a new absorption band at 666 cm^−1^ for lattice vibration of the Mg-O bond and an absorption band related to the O-H stretching vibration of Mg-OH appeared at 3677 cm^−1^, revealing the formation of magnesium silicates [34,35].

Figure 2c,d demonstrate the pore size distribution by the BJH method and the nitrogen adsorption–desorption isotherm by the BET method at 77 K of R-ATP and the prepared composites, and Table 1 lists the corresponding pore parameters. The observation of the Type-IV adsorption isotherm with the H_3_ hysteresis loop exhibited the mesoporous nature of the composite [36,37]. The reaction system can enhance the surface area by adjusting the feeding ratios of MgCl_2_.6H_2_O and the ATP. For example, the synthesized composites had a larger surface area (303–332 m^2^/g), which was over six times larger compared with raw ATP clay (46.15 m^2^/g), which may enhance the adsorption capacity. Compared with raw ATP, pore volume increased from 0.14 cm^3^/g to 0.29 cm^3^/g, while pore sizes decreased from 13.53 nm to 3.74 nm when the Mg/Si ratio reached 0.67. Among the synthesized composites, pore sizes gradually increased from 3.23 nm to 3.74 nm. Consequently, the surface area and porous nature of the materials, which are also important parameters for adsorption, can be adjusted by adjusting the Mg/Si ratios [38,39].

Thermogravimetric analysis was conducted under atmospheric air to study the thermal decomposition of composites. Figure 2e,f display the TGA-DTG curves for the composites. Based on the TGA-DTG curves, thermal degradation of the composite (ATP-MSH-0.67) involved five stages (first (15–156 °C), second (156–264 °C), third (264–424 °C), fourth (424–698 °C), and fifth (698–800 °C)). The release of absorbed water was related to the first stage. The second, third, and fourth stages were attributed to the initial to final releases of hydrated water from the layer of the MSH lattice. On the other hand, the final stage involved the release of water in coordination with magnesium as a result of anhydride formation. Additionally, the hydroxyl group’s breakdown and the release of hydrated water caused a 9.37% mass loss in the synthesized composite.

### 3.2. Surface Morphology

**Figure 3:**
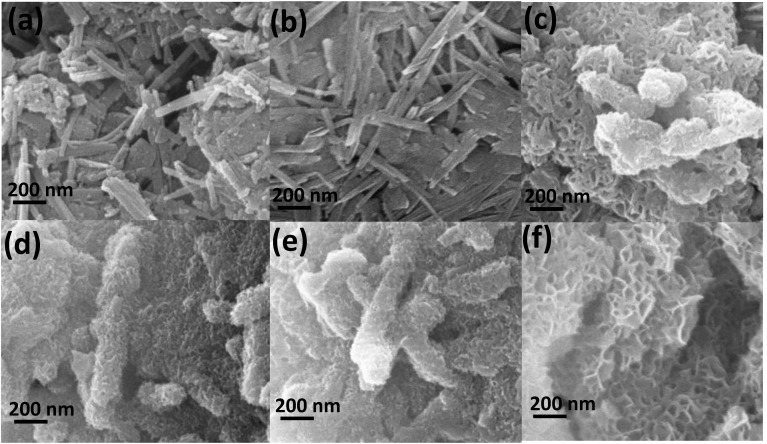
SEM images of (**a**) R-ATP, (**b**) H-ATP, and composites with different Mg/Si ratios: (**c**) 1.50, (**d**) 1.00, (**e**) 0.75, and (**f**) 0.67.

**Figure 4:**
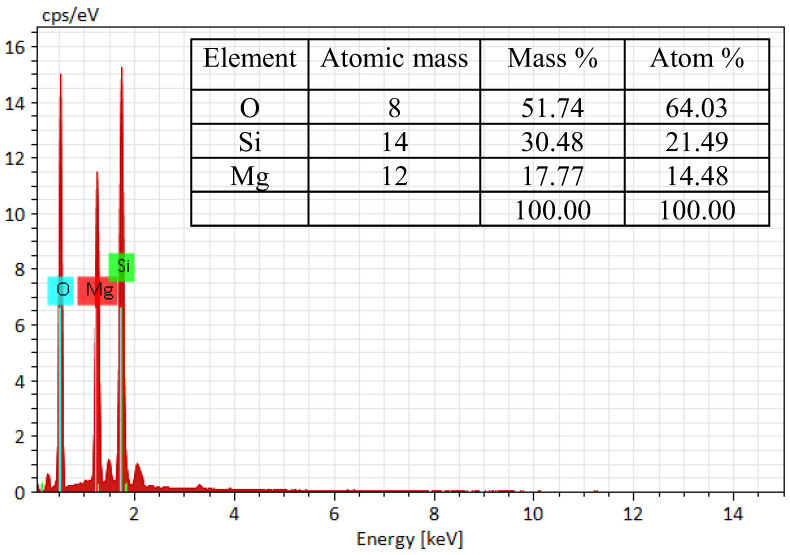
EDS spectrum of the prepared composite (ATP-MSH-0.67).

Figure 3 demonstrates SEM images of raw ATP, acid-leached ATP, and the synthesized composites. Before acid treatment, attapulgite nanofibers (Figure 3a) were clumped together. At the same time, they were split into separate nanofibers or smaller crystal bundles (Figure 3b) after the acid-leaching treatment. Acid attack altered their smooth surfaces into rough ones. After adding different amounts of Mg into the reaction system, honeycomb structures composed of small interlaced nanosheets were formed (Figure 3c–f). Among the synthesized composites, the one with the lowest Mg content (Figure 3f) exhibited the thinnest interlaced sheets along the rod. The morphology of the synthesized products differed noticeably from those of H-ATP and R-ATP, suggesting that the morphological structure of honeycomb composites was significantly influenced by the molar ratio of magnesium to silicon [40]. From the elemental composition of the prepared composite (MSH-ATP-0.67) characterized by EDS spectra, the material was composed of Mg, Si, and O, with corresponding atomic percentages of 14.48%, 21.49%, and 64.03%, showing that the resulted material exhibited the same calculated and experimental Mg-to-Si mole ratio of 0.67 (Figure 4).

### 3.3. Zeta Potential Analysis

**Figure 5:**
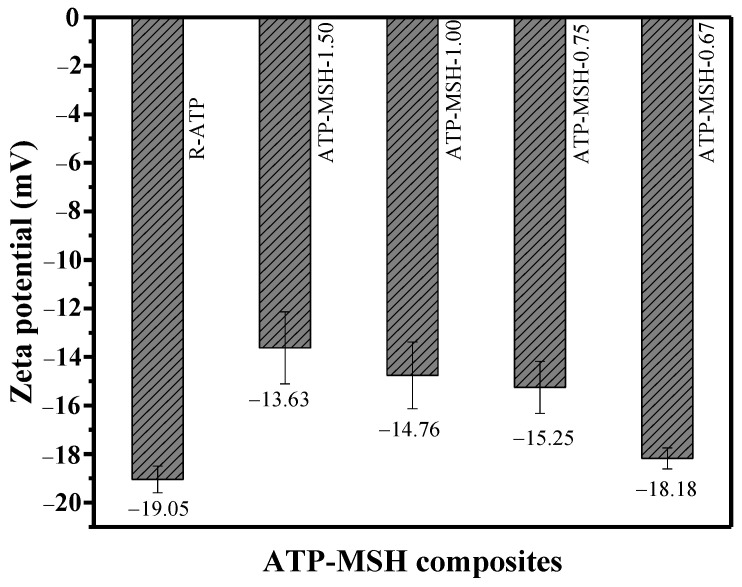
Zeta potential of R-ATP and composites with different Mg/Si ratios.

Figure 5 presents the obtained zeta potential data of R-ATP and composites. Considering the reaction system’s Mg and Si content, the samples with the lowest Mg/Si ratio exhibited a more negative zeta potential compared with the higher Mg/Si ratios [9]. For example, ATP-MSH-1.50 with Mg/Si = 1.50 had a lower zeta potential of −13.63 mV, while ATP-MSH-0.67 with Mg/Si = 0.67 showed a higher zeta potential of −18.18 mV. The higher silanol group was responsible for the material’s increased negative surface charge due to the higher amount of Si than Mg in composites exhibiting better adsorption toward cationic dyes [9]. The most negative charge (−18.18 mV) of ATP-MSH-0.67 with the highest Si content was still less than that (−19.05 mV) of raw ATP, even though the zeta potential of the composites behaved more negatively with higher Si. According to the observed adsorption data, the composite exhibited better MB adsorption results than raw ATP, which could result from the composites’ synergistic effect.

### 3.4. Adsorption of Methylene Blue

**Figure 6:**
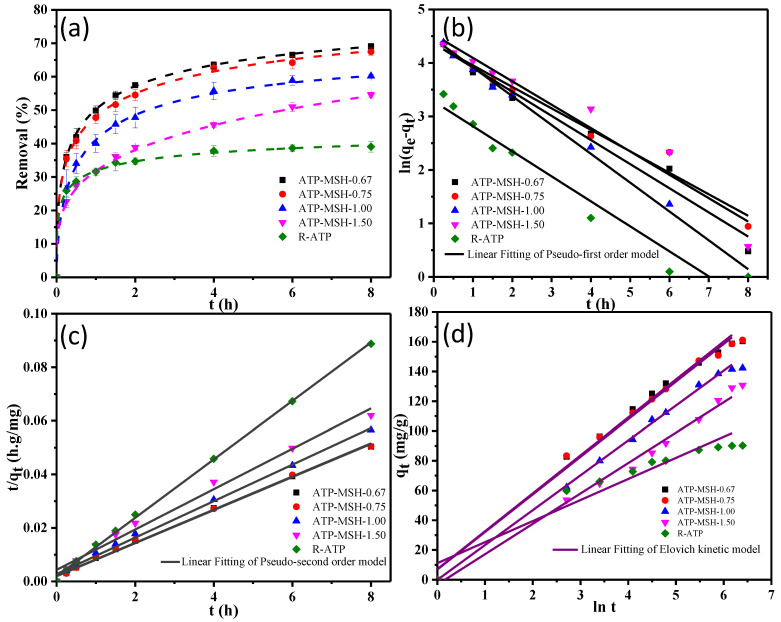
MB adsorption on composites with different Mg/Si ratios and raw ATP for (**a**) removal percentage, (**b**) pseudo-first-order, (**c**) pseudo-second-order, and (**d**) Elovich kinetic models.

The experimental kinetic adsorption results for methylene blue on raw ATP and several composites are shown in Figure 6. Figure 6a displays the effect of contact time on the removal percentage for the adsorption of MB with an initial concentration of 100 mg/L and a 100 mL volume of dye solution on the composite adsorbents with a 0.5 g/L dosage. Although composites had a longer equilibrium time (6 h) compared with raw ATP (1.5 h), they showed better adsorption (54.9–69.1% of MB) than R-ATP (37.8% of MB) toward methylene blue based on the removal percentage, as observed in Figure 6a. Among the tested samples, the sample with the lowest Mg/Si ratio (ATP-MSH-0.67) showed the highest adsorption quantity because of the higher negative surface charge, which is in line with the zeta potential value in Figure 5. Compared with the pseudo-first-order model, the adsorption of MB matched the pseudo-second-order model well, with close values of calculated and experimental adsorption amounts and a relatively higher R^2^ value (Table 2), suggesting that the process was the chemisorption [41]. From Table 3 and Figure 6d, the high R^2^ (0.91–0.99) value in the Elovich kinetic model also revealed that the adsorption was a chemical process [42].

**Figure 7:**
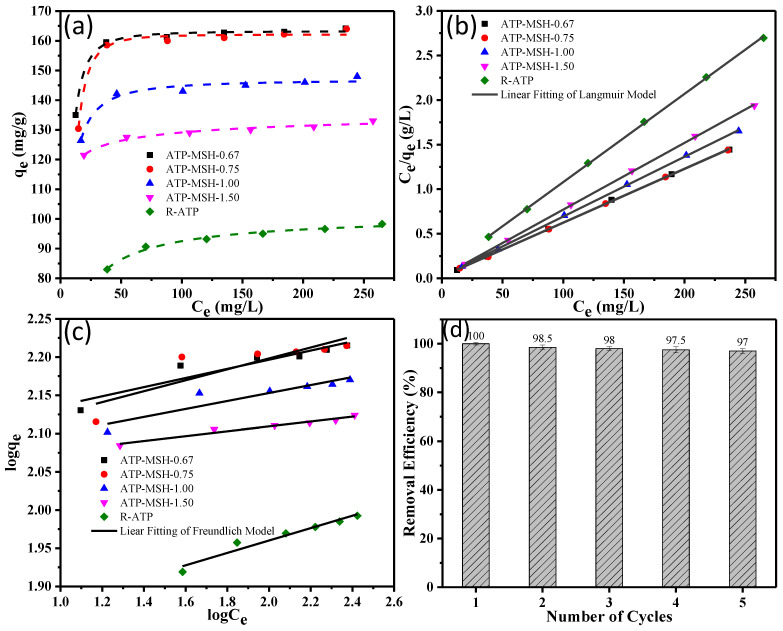
MB adsorption on composites with different Mg/Si ratios and raw ATP for the (**a**) effects of initial dye concentrations, (**b**) Langmuir, (**c**) Freundlich isotherms, and (**d**) recyclability of ATP-MSH-0.67.

Figure 7a demonstrates how the adsorption process was affected by the initial concentration of dye to estimate the maximum adsorption capacity of prepared adsorbents. A strong driving force at higher concentrations caused an increase in adsorption quantity [11]. In Figure 7b,c, the collected adsorption data were linearly fitted to the Freundlich and Langmuir models. Table 4 lists the isotherm parameters. According to the experimental results and the higher linear relativity, the adsorption of MB matched the Langmuir isotherm model notably better than the Freundlich model. It suggests that the adsorption was the monolayer coverage on the surface of composites with equal energy of active sites [43,44]. From the observed data, the assessed maximum adsorption capacities for composites increased with higher Si content (135.14–166.67 mg/g), indicating the prepared composite (ATP-MSH-0.67 with MB adsorption capacity = 166.67 mg/g) showed a better adsorption capacity, 65% higher than that of R-ATP (101.01 mg/g). It was found that the prepared honeycomb composite showed a higher methylene blue adsorption than those documented in the literature (Table 5). Furthermore, the recyclability of ATP-MSH-0.67 was also investigated after regeneration of the adsorbent by calcination at 500 °C for 3 h. From Figure 7d, a removal efficiency of 97% until the fifth cycle revealed that the prepared adsorbent is one of the promising materials for the treatment of textile wastewater.

### 3.5. Adsorption Mechanism

**Figure 8:**
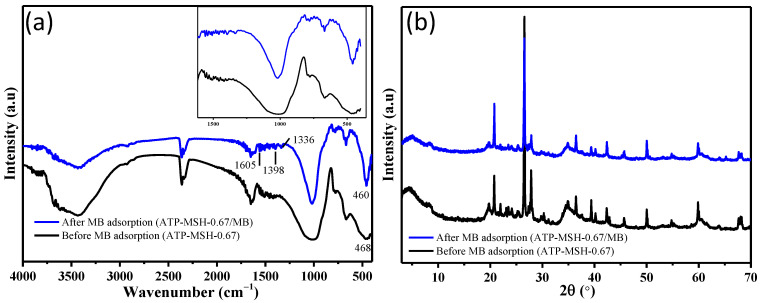
FT-IR spectra (**a**) and XRD patterns (**b**) of the composite (Mg/Si = 0.67) before and after adsorption of methylene blue.

Figure 8a compares FT-IR spectra before and after methylene blue adsorption. Before the adsorption of MB, the vibration bands appeared at 1022 cm^−1^ for the Si-O-Si bond and at 468 cm^−1^ for Mg-O vibration in the FT-IR spectrum of the unmodified composite. Following adsorption, these absorption peaks shifted to new positions. For instance, the Si-O-Si stretching and bending vibrations at 1022 cm^−1^ shifted to 1012 cm^−1^, while the Mg-O vibration band at 468 cm^−1^ moved to 460 cm^−1^, suggesting that the functional groups Mg-O and Si-O may serve as MB’s adsorption sites. After the adsorption of MB, FT-IR spectra displayed the characteristic aromatic ring absorption peak at 1605 cm^−1^ and the C-N stretching vibrations at 1336 and 1398 cm^−1^, indicating the successful adsorption of MB [9]. The adsorption of MB on the MSH composite was indicated by the appearance of broad diffraction peaks of hydrocarbon from MB between 2θ = 20 and 30 degrees, which caused the intensified diffraction peaks of the prepared composite before MB adsorption to decrease in intensity after MB adsorption (Figure 8b). The adsorption of MB suggested that cationic MB adsorbs on porous magnesium silicate through electrostatic attraction with Mg-OH and Si-OH [52].

## 4. Conclusions

Using naturally occurring one-dimensional porous attapulgite as the precursor and a silicon source template, a series of silica porous materials coated with magnesium silicate was prepared under hydrothermal conditions. This work involved the acid-leaching process of extracting silica from clay minerals and optimizing Mg/Si loadings. The composite material with the highest silicon content showed the thinnest interlaced MSH sheets along the rod. The surface area of attapulgite clay was improved by 6.7 times when the magnesium-to-silicon ratio was lowered. After composite formation, the clay’s negative zeta potential of −19.05 mV changed to −18.18 mV. The removal amount for methylene blue by the honeycomb composite was 166.67 mg/g, 65% higher than that of raw ATP clay. Attapulgite may serve as a rod-shaped template and source of silicon for the production of magnesium silicate, as only the surface of the silica was coated with porous magnesium silicate. The synergistic effect of the silica and surface-grown porous magnesium silicate was a main factor of the high removal. This novel approach contributes to the silica-self-templating fabrication of effective porous magnesium silicate adsorbents to remove cationic dye in wastewater treatment.

## Figures and Tables

**Table 1:** Pore parameters of R-ATP and composites with different Mg/Si ratios.

Sample	Surface Area (m^2^/g)	Pore Volume (cm^3^/g)	Average Pore Diameter (nm)
ATP-MSH-0.67	310.41	0.29	3.74
ATP-MSH-0.75	303.14	0.27	3.61
ATP-MSH-1.00	322.48	0.27	3.54
ATP-MSH-1.50	332.57	0.26	3.23
R-ATP	46.15	0.14	13.53

**Table 2:** Kinetic parameters for MB adsorption on composites with different Mg/Si ratios.

Samples	q_e,exp_ (mg/g)	Pseudo-First-Order			Pseudo-Second-Order		
		q_e,cal_ (mg/g)	k_1_ (h^−1^)	R^2^	q_e,cal_ (mg/g)	k_2_ (h^−1^)	R^2^
ATP-MSH-0.67	161.37	91.69	0.209	0.956	163.93	0.018	0.997
ATP-MSH-0.75	161.10	91.10	0.187	0.949	163.93	0.017	0.996
ATP-MSH-1.00	142.30	99.89	0.263	0.985	147.06	0.017	0.996
ATP-MSH-1.50	130.76	89.51	0.159	0.953	136.99	0.012	0.987
R-ATP	90.28	40.36	0.276	0.966	91.74	0.059	0.999

**Table 3:** Elovich kinetic parameters for MB adsorption on composites with different Mg/Si ratios.

Samples	α (mgg^−1^min^−1^)	β (g mg^−1^)	R^2^
ATP-MSH-0.67	35.304	0.040	0.986
ATP-MSH-0.75	34.325	0.040	0.989
ATP-MSH-1.00	24.126	0.044	0.996
ATP-MSH-1.50	17.152	0.048	0.990
R-ATP	5.142	0.074	0.914

**Table 4:** Isotherm parameters for MB adsorption on composites with different Mg/Si ratios.

Samples	Langmuir Model				Freundlich Model		
	q_m_ (mg/g)	b (L/mg)	R^2^	R_L_	K (mg/g)	n	R^2^
ATP-MSH-0.67	166.67	0.0001	0.999	0.96–0.99	119.37	16.72	0.887
ATP-MSH-0.75	166.67	0.0001	0.999	0.96–0.99	113.55	13.99	0.747
ATP-MSH-1.00	149.25	0.0001	0.999	0.95–0.99	111.89	19.19	0.873
ATP-MSH-1.50	133.33	0.0002	0.999	0.95–0.98	111.12	31.45	0.963
R-ATP	101.01	0.0009	0.999	0.78–0.93	62.777	12.315	0.952

**Table 5:** Comparison of MB adsorption for the honeycomb composite (Mg/Si = 0.67) with other adsorbents.

Adsorbents	Adsorption Capacity (mg/g)	Refs.
Luffa fiber	133.58	[45]
NiO-MgO-SBNs	94.7	[46]
Sawdust-based biocarbon	130.98	[47]
Carbon/montmorillonite	138.1	[48]
ZSM-5 zeolite	118.34	[49]
Magnesium silicate	149.3	[50]
Chitosan/montmorillonite	158.7	[51]
ATP-MSH-0.67	166.67	This work

## Data Availability

All data required to evaluate the conclusions of the dissertation are included in the paper. Additional relevant data supporting the findings of this study are available from the corresponding author, Y.J. Feng (yjfeng@mail.buct.edu.cn), upon reasonable request.
